# Retraction Note: Anticancer activity of lactoferrin-coated biosynthesized selenium nanoparticles for combating different human cancer cells via mediating apoptotic effects

**DOI:** 10.1038/s41598-025-94145-4

**Published:** 2025-03-19

**Authors:** Esmail M. El‑Fakharany, Marwa M. Abu‑Serie, Amany Ibrahim, Marwa Eltarahony

**Affiliations:** 1 Protein Research Department, Genetic Engineering and Biotechnology Research Institute (GEBRI), City of Scientific Research and Technological Applications (SRTA-City), New Borg El‑Arab, 21934 Alexandria Egypt; 2 Medical Biotechnology Department, Genetic Engineering and Biotechnology Research Institute (GE‑BRI), City of Scientific Research and Technological Applications (SRTA-City), New Borg El‑Arab, 21934 Alexandria Egypt; 3 Botany Department, Faculty of Women for Arts, Science and Education, Ain Shams University, Cairo, Egypt; 4 Department of Biology, College of Science, Taif University, P.O. Box 11099, 21944 Taif, Saudi Arabia; 5 Ain Shams University, Cairo, Egypt; 6 Environmental Biotechnology Department, Genetic Engineering and Biotechnology Research Institute (GEBRI), City of Scientific Research and Technological Applications (SRTA-City), New Borg El‑Arab, 21934 Alexandria Egypt

Retraction of: *Scientific Reports* 10.1038/s41598-023-36492-8, published online 13 June 2023

The Editors have retracted this Article. After publication, concerns were raised regarding the data presented in Figs. 5 and 6, specifically:

- Fig. 5 ALF-Se NPs Hep-G2 and Caco-2 images appear to overlap;
- The untreated control images in Fig. 5 appear highly similar to those in the authors’ earlier publications (MCF-7, HepG-2 and Caco-2 in Fig. 3b of^1^, and HepG-2 and Caco-2 in Fig. 2b of^2^);
- Fig. 6a ALF-SeNPs plots appear highly similar to Fig. 3 LP-CNPs+LF-FNPs plots in Fig. 3a of^2^.

The Editors therefore no longer have confidence in the presented data.

All Authors disagree with this retraction.
